# Undulatory physical resistance training program increases maximal strength in elderly type 2 diabetics

**DOI:** 10.1590/S1679-45082014AO3162

**Published:** 2014

**Authors:** Gilberto Monteiro dos Santos, Fábio Tanil Montrezol, Luciana Santos Souza Pauli, Angélica Rossi Sartori-Cintra, Emilson Colantonio, Ricardo José Gomes, Rodolfo Marinho, Leandro Pereira de Moura, José Rodrigo Pauli

**Affiliations:** 1 Universidade Federal de São Paulo, Santos, SP, Brazil.; 2 Universidade Estadual de Campinas, Limeira, SP, Brazil.; 3 Faculdade Anhanguera de Campinas, Campinas, SP, Brazil.; 4 Universidade Estadual Paulista “Júlio de Mesquita Filho”, Rio Claro, SP, Brazil.

**Keywords:** Muscular strength, Physical education and training, Aging, Aged, Diabetes mellitus, type 2

## Abstract

**Objective:**

To investigate the effects of a specific protocol of undulatory physical resistance training on maximal strength gains in elderly type 2 diabetics.

**Methods:**

The study included 48 subjects, aged between 60 and 85 years, of both genders. They were divided into two groups: Untrained Diabetic Elderly (n=19) with those who were not subjected to physical training and Trained Diabetic Elderly (n=29), with those who were subjected to undulatory physical resistance training. The participants were evaluated with several types of resistance training’s equipment before and after training protocol, by test of one maximal repetition. The subjects were trained on undulatory resistance three times per week for a period of 16 weeks. The overload used in undulatory resistance training was equivalent to 50% of one maximal repetition and 70% of one maximal repetition, alternating weekly. Statistical analysis revealed significant differences (p<0.05) between pre-test and post-test over a period of 16 weeks.

**Results:**

The average gains in strength were 43.20% (knee extension), 65.00% (knee flexion), 27.80% (supine sitting machine), 31.00% (rowing sitting), 43.90% (biceps pulley), and 21.10% (triceps pulley).

**Conclusion:**

Undulatory resistance training used with weekly different overloads was effective to provide significant gains in maximum strength in elderly type 2 diabetic individuals.

## INTRODUCTION

*Diabetes mellitus *(DM) is a high-incidence disease in Brazil and the world, especially type 2 DM (DM2). This type of diabetes primarily affects adults and the elderly, and has a close relation with obesity. Type 2 DM is a multifactoral condition, characterized by disorders of intermediate metabolism resulting from decreased secretion of insulin and/or decrease in its action (insulin resistance) in peripheral tissues (skeletal muscle and adipose tissue), resulting in hyperglycemia.^(1,2)^ Additionally, other comorbidities may arise along the course of the disease, such as retinopathy, peripheral and autonomic neuropathy, nephropathy, etc. The reduction of muscle mass is a common clinical aspect and is related to the negative protein turnover (proteolysis) in diabetic patients.^(1,2)^

The process of sarcopenia that occurs in senescence is an important aspect that increases the risk of development of insulin resistance and DM2. The etiology of sarcopenia involves various factors, such as loss of motor neurons and cell apoptosis, resulting in a considerable decrease in the number of muscle fibers, especially those for rapid contraction (type II fibers), leading to diminished strength and functional quality of the skeletal muscle.^(3,4)^

The greatest part of the change is associated with aging, and resistance physical exercise can modify this process, or at least mitigate it. Recent scientific evidences are precise in showing that resistance training can prevent the decline in aged-related muscle mass besides maintaining plasticity and capacity for hypertrophy, even during the 10^th^ decade of life, attenuating dynapenia.^(5,6)^

The consequences of skeletal muscle reduction related to aging are diverse, including reduced muscle strength and potency, with a greater frequency of falls and fractures, lower resting metabolic rate, reducing the capacity for oxidizing lipids and an increase in abdominal adiposity. With the increased body fat and physical inactivity, glucose uptake measured by insulin in the skeletal muscle of elderly patients diminished considerably.^(7)^

All these factors contribute towards the loss of autonomy and independence, favoring the development of the metabolic syndrome, with an elevated risk of death by cardiovascular diseases. In this way, maintenance of muscle mass may contribute towards the prevention of disease development, such as obesity, dyslipidemia, and DM2.^(8)^ Various studies showed that the capacity to react to strength training is preserved in elderly individuals and diabetics, with significant gains in physical capacity.^(5,6,9-15)^

Periodization of resistance training or the changes planned in volume and intensity of the exercise are used to maximize the gains in strength and functional conditioning. In this sense, various types of resistance training have been developed. The most common types of training are those with linear (classic) and non-linear (undulatory) characteristics. The big difference between the two plans of work is that in the undulatory periodized training, changes in intensity and volume of the exercise are more frequent and can occur between the days of training, or between the weeks of training.^(16,17)^ In response to resistance training, it is possible to notice an increase in baseline energy expenditure, reduction in body adiposity and lower level of inflammatory process (inflammatory cytokines), and increased glucose uptake by means of increased expression of the glucose type 4 transporter (Glut-4) in skeletal muscle in obese and diabetic individuals.^(9-13,18,19)^ Although both types of training (linear and undulatory) result in increased strength, and improvements in metabolism and functional aptitude, some studies indicate that the results are more positive with undulatory periodization.^(17,19-21)^ Such a fact may be related to the greater stress presumably required with this type of training, and, consequently, more effective neuromuscular adaptations.

As long as our results show positive adaptations in response to undulatory resistance training (URT), new studies are needed with protocols having specific characteristics as to intensity, zone of repetition, and recovery interval to evaluate maximal force, both of lower and upper limbs, especially in elderly type 2 diabetes patients.

## OBJETIVE

To verify the effects of an undulatory physical resistance training protocol on maximal strength gains in elderly type 2 diabetic individuals.

## METHODS

Initially, the study counted on 70 volunteers; however, as per the exclusion criteria, 48 elderly diabetic individuals of both genders, which had entered the multidisciplinary quality of life program developed at the Department Preventive Medicine of Unimed, in the city of Santos, state of São Paulo, remained at the end of the experiment.

All experiments were carried out in the city of Santos during the years 2011 and 2012. The study was performed in accordance with the principles of the Declaration of Helsinki, and was previously submitted to and approved by the Research Ethics Committee of the* Universidade Federal de São Paulo*, under protocol number 0524/11. The volunteers signed an Informed Consent Form before starting the physical training program.

Selection was made by a physician, following the criteria and guidelines established by the American Diabetes Association.^(22)^ Only type 2 diabetic individuals who used antidiabetic drugs and were not dependent on insulin were included, with an age range of 60 to 85 years. The individuals were randomly divided into two groups: Untrained Diabetic Elderly (UDE, n=19), with 13 men; and Trained Diabetic Elderly (TDE, n=29), with 24 women. The participants of the UDE group had the right to be submitted to the same training program soon after the intervention period, with the purpose of offering a physiological response similar to that of the TDE. In order to participate in the project, all individuals declared that they did had not engaged in any type of regular physical activity or supervised exercise in the previous 6 months.

Excluded from the group, whether at selection or during the experimental period, were those individuals who presented with limitations or muscular, joint, or bone diseases; diseases that could compromise the cardiovascular response to the physical training; use of psychotropic substances, such as alcohol and/or other drugs; chronic complications caused by diabetes (autonomic neuropathy, nephropathy, and retinopathy). The volunteers with an attendance under 85% or with three consecutive absences were also excluded. Evasion from the program was greater among male individuals in the TDE. This explains the smaller number of men for this group at the end of the program.

The anthropometric evaluations (weight and height) were performed by a single evaluator, using a digital scales (Filizola^®^ Campo Grande, MS, Brazil), and a stadiometer affixed to the wall (Sanny^®^ Fortaleza, CE, Brazil), as per previous description.^(23)^ From the results obtained, the body mass index (BMI) was calculated. This analysis was only made at the beginning of the experiment in order to establish the profile of the sample studied.

To evaluate blood glucose, a few precautions were taken regarding the procedures, such as: a) the time for collections was between 7:00 am and 8:00 am; b) the participants were kept in fasting state for 12 hours; c) before blood was collected, it was verified that the subjects had not participated in any physical activity on the day before the test; d) all were to remain sitting in a comfortable chair for 10 minutes before the blood was collected; e) the blood was collected by a specialized nurse, using appropriate materials for the procedure. Blood glucose was measured using a specific commercial kit (Laborlab^®^, Paulínia, SP, Brazil), following the manufacturer’s recommendations. Just like with the anthropometric analyses, fasting blood glucose was done only at the beginning of the experiment, in order to establish the profile of the sample studied.

The maximal strength test was done according to the following steps: (1) the participants were familiarized with the equipment during 2 weeks (three sessions/week), using the minimal resistance of the equipment; (2) for the test, the individuals first participated in a warm-up activity, consisting of stretching and performing 20 repetitions with minimal load in the equipment of the test; at the end of the warm-up, the volunteers had 3 minutes of recovery period; (3) next, the 1RM test began, in which the individuals performed two repetitions of the proposed exercise; if they were able to perform it, they had a 5-minute recovery period, and then a new attempt was made with a heavier load; (4) the steps were followed until the moment in which the individuals were able to do only one repetition, thus obtaining the maximal load for each exercise proposed. It is important to emphasize that each person had, at most, five attempts to attain a load regarding the 1RM. When more than five attempts were necessary, the test was performed on another day. The evaluations were done before and after the end of the program, and the last evaluation was performed 72 hours after the last exercise session.

### Undulatory physical resistance training protocol

The diabetic elderly individuals were submitted to resistance exercises on body building equipment or with free weights (dumbbells), lasting 50 minutes, with a weekly frequency of 3 days (Monday, Wednesday, and Friday), with a series prescribed for each exercise, reaching three series along the program (total duration of 16 weeks).

During the first week, the volunteers performed the physical training initiating with a load equivalent to 50%, with one series on Monday, two on Wednesday, and three series on Friday. During the second week, training began with a load equivalent to 70%, with one series on Monday, two on Wednesday, and three series on Friday. From the third week on, the three series were maintained for each exercise, alternating each week of the work load (50% on odd weeks – 1^st^, 3^rd^, 5^th^, 7^th^, 9^th^, 11^th^, 13^th^, and 15^th^ week - and 70% on the even weeks – 2^nd^, 4^th^, 6^th^, 8^th^, 10^th^, 12^th^, 14^th^, and 16^th^ week). The equipment used was Nakagin (SP, Brazil).

Periodization of training was based on the recommendation of progressive strength training for initiating adults and type 2 diabetics^(24,25)^. In this way, the protocol consisted of a weekly alteration of the intensity divided into a week of moderate overload (70% of 1RM, 8 repetitions) and a week of light overload (50% of 1RM, 12 repetitions).

**Figure 1:**
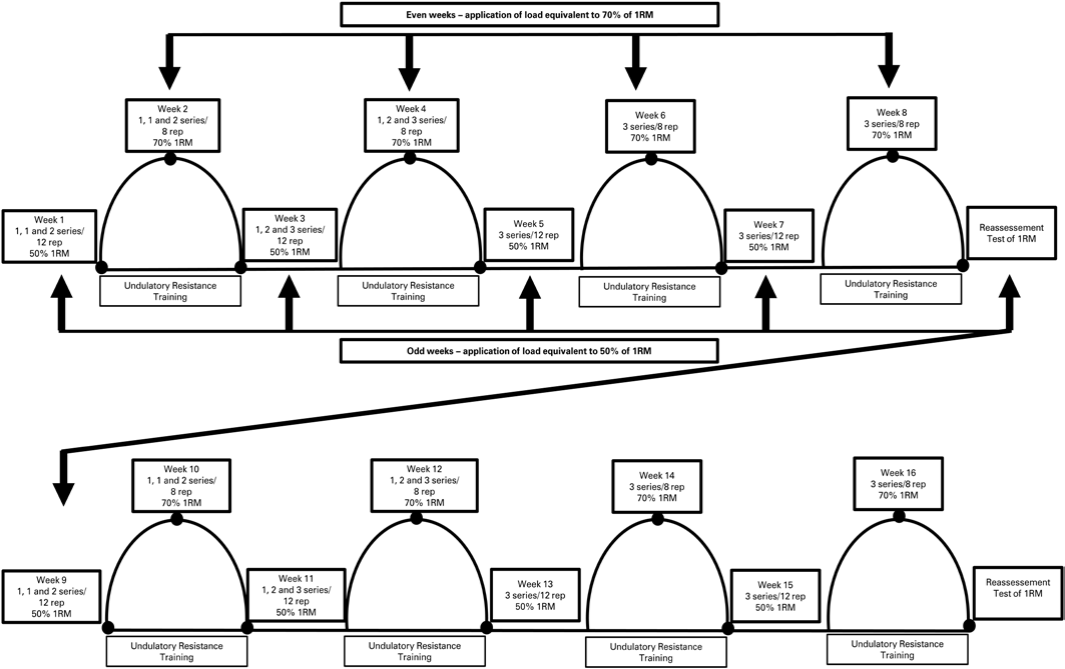
Experimental design of undulatory periodization. 1RM: test with one maximal repetition; Rep: repetitions

Figure 1 shows the model of URT used. The interval between the series depended on the load adopted at the training session, with 2-minute intervals for the weeks with moderate loads and one minute for the weeks with light loads.

Ten exercises were selected, working both the agonist and antagonist muscles of each movement, without provoking muscular imbalance. Exercises for the abdominal and lumbar regions were not forgotten, since these are essential for stabilization and balance of many movements. The muscle groups shown on chart 1 were evaluated and trained. Also included were the following exercises: sitting development with dumbbells, standing plantar flexion, partial abdominal exercises, and lumbar extension. The latter exercises were not evaluated by the difficulty of performing the maximal load test or adjustments during training.

**Chart 1:** Type of exercise and muscles involved in the program of physical resistance training used in evaluating the test with one single maximal repetition

Type of exercise	Muscles involved
Knee extension (sitting)	Quadriceps (vastus lateralis, vastus intermedius, vastus medialis, and rectus femoralis)
Knee flexion (lying down)	Ischiotibials (semimembranosus, semitendinosus, and femoral biceps)
Straight supine (sitting)	Pectoralis major, pectoralis minor, deltoid (clavicular portion), serratus anterior, and brachial triceps
Straight rowing (sitting)	Latissimus dorsi, trapezius (transverse portion), rhomboids, teres major, deltoid (spinal portion), brachial biceps, brachial, and brachioradial
Biceps pulley (standing)	Brachial biceps, brachia, and brachioradial
Triceps pulley (standing)	Brachial triceps

Work load adjustment was made throughout the time that the subject performed with the established load, 15 repetitions in training at 50% of 1RM (going back to the 12 repetitions) and 12 repetitions in training at 70% of 1RM (going back to the 8 repetitions). The UDE group received no intervention and was instructed to not change their lifestyle habits during this period of the physical training protocol.

### Statistical analysis

Initially, all data were submitted to the Kolmogorov-Smirnov test, with the purpose of determining if their distributions of probability presented as parametric or non-parametric. All the data showed normal distribution. Values were expressed as mean ± standard deviation (SD). To compare the behaviors of the TDE and UDE groups along time, according to each variable of interest, the model of variance analysis (ANOVA) with repeated measurements was used, followed by Bonferroni’s post-testing. The values were considered statistically significant when p<0.05. For all these procedures, the GraphPad Prism statistical software, version 3.02 (GraphPad Software, San Diego, CA, USA) was used.

## RESULTS

The results in reference to characterization of the sample were extracted from the databank of the participants in the multidisciplinary quality of life program, of the Preventive Medicine Sector of Unimed, in the city of Santos. The anthropometric variables evaluated obtained parametric distribution for both groups (age, body mass, stature, and BMI). The BMI indicated that the participants were within the range considered as pre-obese, with an increased risk of comorbidities, according to the World Health Organization (WHO).^(26)^ Capillary glucose demonstrated that all individuals presented with altered glycemic levels, which is characteristic of type 2 diabetic individuals (Table 1).

**Table 1:** Anthropometric and fasting glucose characteristics of the groups of Untrained and Trained Diabetic Elderly at baseline

Groups/variables	UDE (n=19)	TDE (n=29)
Age (years)	66.30±4.74	66.87±5.36
Weight (kg)	78.09±9.58	73.48±12.32
Height (m)	1.64±0.08	1.60±0.06
BMI (kg/m^2^)	29.03	28.70
Fasting capillary glucose (mg/dL)	156.56±21.18	152.26±26.14

BMI: body mass index; UDE: untrained diabetic elderly; TDE: trained diabetic elderly.

In the 1RM test, the statistical analysis revealed significant differences between the pre- and post-period results of the TDE intervention group. The participants in this group obtained significant gains in maximal strength in all exercises performed (knee extension and flexion, supine, and triceps and biceps pulley) (Table 2). These results indicate that the URT was effective in increasing the maximal strength in elderly diabetics after 16 weeks of intervention. Such a fact, however, was not observed in the UDE group.

**Table 2:** Data obtained from the test with a single maximal repetition of each exercise, as per group and time

Groups/variables	UDE (n=19)		TDE (n=29)		Increased of maximal strength (TDE group) %
	Pre-	Post-	Pre-	Post-	
Lower limbs (kg)					
Knee extension (sitting)	8.05±2.04	8.85±2.58	11.65±2.69	16.68±3.04* (p<0.001)	43.2
Knee flexion (lying down)	7.55±0.51	7.55±1.57	8.19±1.76	13.52±2.25* (p<0.001)	65.1
Upper limbs (kg)					
Chest press	20.85±3.60	21.35±3.72	18.58±2.62	23.74±2.82* (p<0.001)	27.8
Sitting rowing	22.10±4.27	22.75±3.58	19.19±3.89	25.13±4.43* (p<0.001)	31
Triceps pulley	21.00±4.97	21.95±5.04	20.16±2.19	24.42±2.68* (p<0.001)	21.1
Biceps pulley	6.55±1.76	7.05±1.61	5.45±0.96	7.84±1.32* (p<0.001)	43.9

*Difference between pre- and post-training condition (p<0.001). Data expressed as means and ± standard deviation. UDE: untrained diabetic elderly; TDE: trained diabetic elderly.

## DISCUSSION

In an attempt of intervention and health improvement, especially an increase in maximal strength, which might have repercussions in terms of positive changes in the body, such as increased autonomy, independence, and metabolic changes,^(18)^ in the present study an URT protocol was used with changes in weekly intensity and volume. As to elderly diabetic patients, the mean increases in strength for each muscle group evaluated in the study were very significant.

The biggest mean increase in strength was observed for the movement of knee flexion (65.1%), followed by movements of knee extension and biceps pulley (43.2% and 43.9%), respectively. The movements of knee extension and knee flexion are fundamental in daily life activities for squatting, rising, and in moving around, and are of vital importance for this population. The increased strength of the muscles involved in elbow flexion (biceps pulley) of 43.9% relative to the pre-training condition was able to help the individuals in performing their daily tasks and work which require the use of upper limbs such as, for example, transporting a bag of purchases from the grocery store, hanging clothes on a line to dry and then remove them, carry objects, etc. It seems that this increased strength in elbow flexor muscles is related to the fact that the brachial biceps muscles, brachial, and radial muscles participate in the movement both in the biceps pulley and in sitting rowing, which could justify, a least in part, this increased gain in strength.

The effects of the process of aging, associated with physical inactivity, act in different manners on the upper and lower limbs. The decline in strength seems to be much more accentuated in the lower limbs.^(27-30)^ In this way, it is expected that for sedentary individuals, the response to physical strength training is more evident in the lower limbs, since these are the ones least trained. Since in the present study there were participants who were not physically active elderly individuals, this response to training was observed, revealing an increase in strength greater for the lower limbs.

In the other exercises performed, also verified were mean increases in strength for the movement of chest press (27.8%) and triceps pulley (21.1%). Such a fact demonstrates that the physical training developed was efficient in increasing the strength of the participants. Nevertheless, an even more accentuated increase was expected in this variable for the movement of forearm extension, since in the chest press and in dumbbells shoulder press, the triceps muscle also participates in putting forth strength, resulting, therefore, in a sum of recruited muscles in the movement.

The particularity of this study was in the proposal of an URT protocol, with a frequency of three times a week, with different intensity and volume each weak, performed in a periodic manner (load increase). This type of training with the objective of increasing the maximal strength of elderly diabetic individuals has been explored very few times in literature. When compared to the various protocols of physical strength training used in type 2 diabetics, the physical training we propose proved very efficient in promoting increased strength.^(8-12)^ Studies in literature performed with other populations (children and young adults), have also observed more significant responses in maximal strength and metabolic parameters (for example: insulin sensitivity) with URT relative to linear resistance training.^(17,21)^

In our study, the mean increase in strength of the lower limbs was 54.15%, and of the upper limbs, 30.95% in 16 weeks of physical training; these results are similar or superior to those found in literature.^(8-12,23)^ However, one needs to mention that such a fact may be related to the characteristic of the sample, which consisted of diabetic elderly individuals, who were not physically active, and unfamiliar with strength training. According to the principles of training, physically trained individuals (i.e., highly fit) presented with lower adaptive responses to a physical exercise program.^(31)^

Dunstan et al.,^(32)^ based on resistance physical training with progressive loads, initiating with 50% and ending the training period with loads reaching 80% of 1RM, with a duration of physical exercise program of 6 months, obtained results similar to those found in this study as to strength gain in elderly diabetic individuals. However, different from the present study, the greatest strength gain was obtained in the upper limbs of trained elderly diabetics.^(32)^ On the other hand, Cauza et al.,^(14)^ when using a program of physical resistance training with increased intensity and volume with a progressive increase of working loads and a duration of 16 weeks, in a population of adults with DM2, also observed good responses to strength training in this specific population. Similar to the results of the present study, Cauza et al. also observed greater increases in strength in lower limbs when compared to the upper limbs.^(14)^

In another study, also with a population of older diabetic women, with a mean age of 68 years, Guido et al. obtained results similar to those of the present study, observing that resistance training for 24 weeks, with progressive intensities every 4 weeks, initiating at 60% and reaching up to 80% of 1RM, was capable of increasing the strength of lower limbs, especially of the extensor muscles of the knee.^(33)^ Although the results obtained in literature point to increased strength with linear training, one of the positive aspects observed in URT is that the execution (routine) of the exercises becomes less monotonous and therefore, there is greater attendance of the program participant.^(16)^

The increase in the strength obtained with resistance physical training in the present study may result in better quality of life and in autonomy of the participants. Strength is a very important physical capability, which, when increased, also affords improvement of other capacities, such as agility and balance, which is extremely important in order to avoid accidents at home in senior citizens.^(34)^ Ferreira and Gobbi^(35)^ verified that active older women, who consequently have better levels of strength, show better levels of agility in the lower limbs when compared to sedentary older women.

Taking into consideration that with aging there is the so-called “sarcopenia” phenomenon, which is the decrease of muscle mass, and that the capacity of the muscle to generate strength in human beings declines especially after 60 years of age,^(36)^ one can say that resistance training performed by the participants contributed towards retarding this process, allowing additional gains in strength throughout 16 weeks. However, new studies are needed with active diabetic individuals and for a greater period of time, in order to evaluate the results of the program proposed in this study.

## CONCLUSION

According to the sample studied, and taking into consideration the limitations of the present study (smaller number of female individuals in the group of Untrained Diabetic Elderly relative to the group Trained Diabetic Elderly, besides the short intervention period – 16 weeks), it was possible to conclude that the undulatory resistance physical training protocol used proved efficient in providing significant increases in maximal strength, both in lower and upper limbs in type 2 diabetic individuals who are not physically active. We suggest that the program proposed herein be used as a different alternative in strength training for the aged type 2 diabetic population, and especially, in reference to healthcare professionals who treat this specific population.
