# Supplementary material for: Undulatory physical resistance training program increases maximal strength in elderly type 2 diabetics
Source: Einstein (Sao Paulo). 2014 Oct-Dec;12(4):425–32. doi: 10.1590/S1679-45082014AO3162 (PMC4879907; doi:10.1590/S1679-45082014AO3162)
Supplement: Translation

# Translation: Programa de treinamento físico resistido ondulatório aumenta a força máxima de idosos diabéticos tipo 2

**Authors:** Gilberto Monteiro dos Santos, Fábio Tanil Montrezol, Luciana Santos Souza Pauli, Angélica Rossi Sartori-Cintra, Emilson Colantonio, Ricardo José Gomes, Rodolfo Marinho, Leandro Pereira de Moura, José Rodrigo Pauli

**Affiliations:** 1 Universidade Federal de São Paulo, Santos, SP, Brasil.; 2 Universidade Estadual de Campinas, Limeira, SP, Brasil.; 3 Faculdade Anhanguera de Campinas, Campinas, SP, Brasil.; 4 Universidade Estadual Paulista “Júlio de Mesquita Filho”, Rio Claro, SP, Brasil.

**Keywords:** Força muscular, Educação física e treinamento, Envelhecimento, Idoso, Diabetes mellitus tipo 2

## Abstract

**Objetivo:**

Verificar os efeitos de um protocolo de treinamento físico resistido ondulatório nos ganhos de força máxima em idosos diabéticos do tipo 2.

**Métodos:**

Participaram do estudo 48 indivíduos, com idade entre 60 e 85 anos, de ambos os gêneros. Eles foram divididos em dois grupos: Idosos Diabéticos Não Treinados (n=19), com aqueles não submetidos ao treinamento físico, e Idosos Diabéticos Treinados (n=29), que foram submetidos ao protocolo de treinamento físico resistido ondulatório. Os idosos foram avaliados em diversos equipamentos de musculação, antes e após o treinamento resistido ondulatório, por meio do teste de uma repetição máxima. Os participantes realizaram o treinamento resistido ondulatório três vezes por semanas, durante um período de 16 semanas. A sobrecarga do programa foi alternada, sendo em 1 semana equivalente a 50% de uma repetição máxima e, na outra semana, a 70% de uma repetição máxima. A análise estatística revelou diferenças significativas (p<0,05) entre os resultados dos testes pré e pós-período de treinamento resistido ondulatório em um período de 16 semanas.

**Resultados:**

Os ganhos médios de força foram de 43,20% (extensão de joelho), 65,00% (flexão de joelho), 27,80% (supino sentado máquina), 31,00% (remada sentado), 43,90% (bíceps *pulley*) e 21,10% (tríceps *pulley*).

**Conclusão:**

O protocolo de treinamento resistido ondulatório utilizado com sobrecargas semanais diferentes foi eficiente em proporcionar significativos ganhos de força máxima em idosos diabéticos do tipo 2.

## INTRODUÇÃO

O *diabetes* *mellitus *(DM) é uma doença de alta incidência no Brasil e no mundo, principalmente o DM do tipo 2 (DM2). Esse tipo de diabete acomete principalmente adultos e idosos, e tem estreita relação com obesidade. O DM do tipo 2 é uma doença multifatorial, caracterizada por distúrbios no metabolismo intermediário, decorrentes da diminuição na secreção da insulina e/ou diminuição na sua ação (resistência à insulina) em tecidos periféricos (músculo esquelético e tecido adiposo), resultando em hiperglicemia.^(1,2)^ Ademais, outras comorbidades podem surgir ao longo do curso da doença, como a retinopatia, a neuropatia periférica e autonômica, a a nefropatia etc. A redução da massa muscular é um aspecto clínico comum e que está atrelado ao *turnover* proteico negativo (proteólise) nos pacientes diabéticos.^(1,2)^

O processo de sarcopenia, que acontece na senescência, é um aspecto importante e que aumenta o risco de desenvolvimento de resistência à insulina e de DM2. A etiologia da sarcopenia envolve diversos fatores, como perda de neurônios motores e apoptose celular, tendo como consequência a diminuição considerável do número de fibras musculares, em especial as de contração rápida (fibras do tipo II), induzindo ao decréscimo da força e da qualidade funcional do músculo esquelético.^(3,4)^

A maior parte das mudanças está associada com o avançar da idade, e o exercício físico resistido pode modificar esse processo ou, pelo menos, atenuá-lo. Recentes evidências científicas são precisas em mostrar que o treinamento resistido pode prevenir o declínio na massa muscular relacionada à idade, além de manter a plasticidade e a capacidade de hipertrofia, mesmo na 10^a ^década de vida, atenuando a dinapenia.^(5,6)^

As consequências da redução do músculo esquelético relacionadas ao envelhecimento são diversas, incluindo redução da força e potência muscular, com maior frequência de quedas e fraturas, menor taxa metabólica de repouso, reduzida capacidade para oxidação de lipídios e aumento da adiposidade abdominal. Com o aumento do conteúdo de gordura corporal e inatividade física, a captação de glicose mediada por insulina no músculo esquelético de pacientes idosos diminui consideravelmente.^(7)^

Todos esses fatores contribuem para a perda de autonomia e independência, favorecendo o desenvolvimento da síndrome metabólica, com elevado risco de morte por doenças cardiovasculares. Desse modo, a manutenção da massa muscular pode contribuir para a prevenção de desenvolvimento de doenças, como obesidade, dislipidemia e o DM2.^(8)^ Diversos estudos mostraram que a capacidade para reagir ao treinamento de força está preservada em indivíduos idosos e diabéticos, sendo significativos os ganhos nessa capacidade física.^(5,6,9-15)^

A periodização do treinamento resistido, ou as mudanças planejadas no volume e na intensidade do exercício são utilizadas para maximizar os ganhos de força e aptidão funcional. Nesse sentido, diversos tipos de treinamento resistido têm sido desenvolvidos. Os tipos mais comuns de treino resistido são os de característica linear (clássico) e o não linear (ondulatório). A grande diferença entre os dois planos de trabalho é que, na periodização do treinamento ondulatório, ocorrem mudanças na intensidade e no volume de exercício com mais frequência, podendo ocorrer entre os dias de treinamento, ou variar entre as semanas de treino.^(16,17)^ Em reposta ao treinamento resistido, podem-se observar aumento do gasto energético basal, redução da adiposidade corporal e do processo inflamatório de baixo grau (citocinas inflamatórias), aumento na captação de glicose por meio do aumento da expressão do transportador de glicose tipo 4 (Glut-4) no músculo esquelético em pessoas obesas e diabéticas.^(9-13,18,19)^ Embora ambos os tipos de treinamento (linear e ondulatório) resultem em aumentos de força, melhoras metabólicas e de aptidão funcional, algumas pesquisas indicam que os resultados são mais positivos com a periodização ondulatória.^(17,19-21)^ Tal fato pode estar relacionado ao maior estresse presumidamente requerido nesse tipo de treinamento e, consequentemente, adaptações neuromusculares mais efetivas.

Conquanto nossos resultados mostrem adaptações positivas em resposta ao treinamento resistido ondulatório (TRO), são necessários novos estudos com protocolos de características específicas quanto à intensidade, zona de repetição e intervalo de recuperação, para avaliação da força máxima, tanto de membros inferiores, quanto de membros superiores especialmente em idosos diabéticos do tipo 2.

## OBJETIVO

Verificar os efeitos de um protocolo de treinamento físico resistido ondulatório nos ganhos de força máxima em idosos diabéticos do tipo 2.

## MÉTODOS

Inicialmente, o estudo contou com 70 indivíduos voluntários; contudo, obedecendo aos critérios de exclusão, permaneceram até ao final do experimento, 48 idosos voluntários diabéticos de ambos os gêneros, ingressantes no programa multidisciplinar de qualidade de vida, desenvolvido na seção de Medicina Preventiva da Unimed de Santos, no Estado de São Paulo.

Todos os experimentos foram realizados na cidade de Santos, nos anos de 2011 e 2012. O estudo foi realizado de acordo com os princípios da declaração de Helsinki, sendo previamente submetido e aprovado pelo Comitê de Ética e Pesquisa da Universidade Federal de São Paulo, com protocolo 0524/11. Os voluntários assinaram um Termo de Consentimento Livre Esclarecido antes de iniciar o programa de treinamento físico.

Foi realizada uma triagem por um médico, que obedeceu aos critérios e diretrizes estabelecidos pela Associação Americana de Diabetes.^(22)^ Apenas foram selecionados os indivíduos diabéticos do tipo 2 com o uso de hipoglicemiantes e não dependentes de insulina, dentro da faixa etária de 60 a 85 anos. Os indivíduos foram aleatoriamente divididos em dois grupos: Idosos Diabéticos Não Treinados (IDNT, n=19), sendo 13 homens; e Idosos Diabéticos Treinados (IDT, n=29), sendo 24 mulheres. Os participantes do grupo IDNT tiveram o direito de serem submetidos ao mesmo programa de treinamento, logo após o período de intervenção, com intuito de oferecer a resposta fisiológica similar ao IDT. Para participar do projeto, todos os indivíduos afirmaram que não realizaram nenhum tipo de atividade física regular ou exercício supervisionado nos últimos 6 meses.

Foram excluídos do estudo, quando na triagem, ou durante o período experimental, os indivíduos que apresentaram limitações ou doenças musculares, articulares ou ósseas; doenças que pudessem comprometer a resposta cardiovascular ao treinamento físico; uso de substâncias psicotrópicas, como álcool e/ou outras drogas; complicações crônicas causadas pelo diabetes (neuropatia autonômica, nefropatia e retinopatia). Os voluntários que tiveram assiduidade menor que 85% ou três ausências seguidas também foram excluídos. A evasão do programa foi maior entre os indivíduos do gênero masculino do grupo IDT. Isso explica o menor número de homens para esse grupo ao final do programa.

As avaliações antropométricas (massa corporal e estatura) foram realizadas por um único avaliador, utilizando-se balança digital (Filizola^®^ Campo Grande, MS, Brasil) e estadiômetro fixo na parede (Sanny^®^ Fortaleza, CE, Brasil), conforme descrição prévia.^(23)^ A partir dos resultados obtidos, foi calculado o índice de massa corporal (IMC). Essa análise foi realizada apenas no início do experimento, para estabelecer o perfil da amostra estudada.

Para avaliação da glicemia, foram tomados alguns cuidados quanto aos procedimentos, como: a) o horário da coleta foi entre às 7 e 8h; b) os participantes tiveram que ficar de jejum por 12 horas; c) antes de realizar a coleta de sangue, foi certificado que os participantes não tinham realizado nenhuma atividade física no dia anterior do exame; d) todos tiveram que permanecer por 10 minutos sentados em uma cadeira confortável antes da coleta; e) a coleta do sangue foi realizada por enfermeira especializada, utilizando-se de materiais apropriados para o procedimento. A glicemia foi mensurada por meio de *kit *específico comercial (Laborlab^®^, Paulínia, SP, Brasil), seguindo as recomendações do fabricante. Assim como as análises antropométricas, a glicemia de jejum foi realizada apenas no início do experimento, para estabelecer o perfil da amostra estudada.

O teste de força máxima foi realizado conforme os seguintes passos: (1) os participantes foram familiarizados com os equipamentos durante 2 semanas (três sessões/semana), utilizando-se da resistência mínima dos equipamentos; (2) para o teste, os indivíduos realizaram previamente aquecimento, que consistiu de alongamento executado no próprio equipamento de musculação (20 repetições com carga mínima); ao final do aquecimento, foi concedido um período de recuperação de 3 minutos; (3) em seguida, iniciou-se o teste de 1RM, no qual os indivíduos realizaram duas repetições do exercício proposto; caso conseguissem realizá-las, era concedido um prazo de 5 minutos para recuperação e, então, uma nova tentativa era realizada com uma carga maior; (4) os passos foram seguidos até o momento em que os indivíduos realizassem apenas uma repetição, obtendo-se, então, a carga máxima para cada exercício proposto. Vale ressaltar que cada indivíduo teve, no máximo, cinco tentativas para se obter a carga referente a 1RM. Quando necessário mais de cinco tentativas, o teste era realizado em outro dia. As avaliações foram realizadas antes e após o término do programa, sendo que a última avaliação foi realizada após 72 horas da última sessão de exercício.

### Protocolo de treinamento físico resistido ondulatório

Os idosos diabéticos foram submetidos a exercícios resistidos em equipamentos de musculação ou com pesos livres (halteres), de duração de 50 minutos, com frequência semanal de 3 dias (segunda, quarta e sexta-feira), sendo prescrita, no início, uma série para cada exercício, atingindo três séries no decorrer do programa (duração total de 16 semanas).

Na primeira semana, os voluntários realizaram o treinamento físico iniciando com a carga equivalente a 50%, sendo realizadas uma série na segunda-feira, duas na quarta-feira e três séries na sexta-feira. Na segunda semana, o treinamento iniciou com a carga equivalente a 70%, sendo realizadas uma série na segunda-feira, duas na quarta-feira e três séries na sexta-feira. Da terceira semana em diante, foram mantidas as três séries para cada exercício, sendo em cada semana alternada a carga de trabalho (50% nas semanas ímpares − 1ª, 3ª, 5ª, 7ª, 9ª, 11ª, 13ª e 15ª semana − e 70% nas semanas pares − 2ª, 4ª, 6ª, 8ª, 10ª, 12ª, 14ª e 16ª semana). Os equipamentos utilizados foram da marca Nakagin (SP, Brasil).

A periodização do treinamento foi baseada nas recomendações do treinamento de força progressivo para adultos iniciantes e indivíduos diabéticos do tipo 2.^( ^24^ , ^25^ )^ Dessa forma, o protocolo consistiu na alteração semanal da intensidade dividida em semana de sobrecarga moderada (70% de 1RM, 8 repetições) e semana de sobrecarga leve (50% de 1RM, 12 repetições).

Na figura 1, está representado o modelo de TRO utilizado. O intervalo entre as séries dependeu da carga adotada na sessão de treinamento, tendo intervalos de 2 minutos para as semanas com cargas moderadas e de um minuto para as semanas com cargas leves.

**Figura 1:** Desenho experimental da periodização ondulatória. 1RM: teste de uma repetição máxima; Rep: repetições

Foram selecionados dez exercícios, sendo trabalhados tanto os grupos musculares agonistas quanto antagonistas de um movimento, sem provocar desequilíbrio muscular. Não foram esquecidos os exercícios para a região abdominal e lombar, pois esses são essenciais para estabilização e equilíbrio de muitos movimentos. Foram avaliados e treinados os grupos musculares dispostos no quadro 1. Houve também a inclusão dos seguintes exercícios: desenvolvimento sentado com halteres, flexão plantar em pé, abdominal parcial e extensão lombar. Esses últimos não foram avaliados pela dificuldade de realizar o teste de carga máxima bem como de ajustes nas cargas ao longo do treinamento.

**Quadro 1:** Tipo de exercício e músculos envolvidos no programa de treinamento físico resistido utilizado na avaliação de teste de uma repetição máxima

Tipo de exercício	Músculos envolvidos
Extensão dos joelhos (sentado)	Quadríceps (vasto lateral, vasto intermédio, vasto medial e reto femoral)
Flexão dos joelhos (deitado)	Isquiotibiais (semimenbranoso, semitendinoso e bíceps femoral)
Supino reto (sentado)	Peitoral maior, peitoral menor, deltoide (parte clavicular), serrátil anterior e tríceps braquial
Remada reto (sentado)	Latíssimo do dorso, trapézio (parte transversal), romboides, redondo maior, deltoide (parte espinal), bíceps braquial, braquial e braquiorradial
Bíceps *pulley* (rosca bíceps em pé)	Bíceps braquial, braquial e braquiorradial
Tríceps *pulley* (rosca tríceps em pé)	Tríceps braquial

O ajuste da carga de trabalho foi realizado à medida que o sujeito realizava, com a carga estabelecida, 15 repetições no treino a 50% de 1RM (retornando para as 12 repetições) e 12 repetições no treino a 70% de 1RM (retornando para as 8 repetições). O grupo IDNT não recebeu nenhuma intervenção e foi orientado a não alterar seus hábitos de vida nesse período de execução do protocolo de treinamento físico.

### Análise estatística

Inicialmente, todos os dados foram submetidos ao teste de Kolmogorov-Smirnov, com o intuito de determinar se suas distribuições de probabilidade apresentavam-se como paramétricas ou não paramétricas. Todos os dados apresentaram uma distribuição normal. Os valores foram expressos como a média ± desvio padrão (DP). Para comparar o comportamento dos grupos IDT e IDNT ao longo do tempo, segundo cada variável de interesse, foi empregado o modelo de análise de variância (ANOVA) com medidas repetidas, seguidas do pós-teste de Bonferroni. Os valores foram considerados estatisticamente significativos quando p<0,05. Para todos esses procedimentos, foi utilizado o programa estatístico *GraphPad Prism* versão 3.02 (*GraphPad Software, San Diego*, CA, Estados Unidos).

## RESULTADOS

Os resultados referentes à caracterização da amostra foram extraídos do banco de dados dos participantes do programa multidisciplinar de qualidade de vida, da Seção de Medicina Preventiva da Unimed de Santos. As variáveis antropométricas avaliadas obtiveram distribuição paramétrica para ambos os grupos (idade, massa corporal, estatura e IMC). O IMC indicou que os participantes estavam na faixa considerada de pré-obeso, com risco aumentado de comorbidades, segundo Organização Mundial de Saúde (OMS).^(26)^ A glicemia capilar demonstrou que todos os indivíduos apresentaram níveis glicêmicos alterados, característicos de indivíduos diabéticos do tipo 2 (Tabela 1).

**Tabela 1:** Características antropométricas e de glicemia de jejum dos grupos de Idosos Diabéticos Não Treinados e Treinados no início do estudo

Grupos/variáveis	IDNT (n=19)	IDT (n=29)
Idade (anos)	66,30±4,74	66,87±5,36
Massa corporal (kg)	78,09±9,58	73,48±12,32
Estatura (m)	1,64±0,08	1,60±0,06
IMC (kg/m^2^)	29,03	28,70
Glicemia capilar de jejum (mg/dL)	156,56±21,18	152,26±26,14

IMC: índice de massa corporal; IDNT: idosos diabéticos não treinados; IDT: idosos diabéticos treinados.

No teste de 1RM, a análise estatística revelou diferenças significativas entre os resultados pré e pós-período de intervenção no grupo IDT. Os participantes desse grupo obtiveram ganhos significativos de força máxima em todos os exercícios realizados (extensão e flexão de joelhos, supino e tríceps e bíceps *pulley*) (Tabela 2). Esses resultados indicam que o TRO foi efetivo em aumentar a força máxima nos idosos diabéticos após 16 semanas de intervenção. Tal fato, porém, não foi observado para o grupo IDNT.

**Tabela 2:** Dados obtidos no teste de uma repetição máxima em cada exercício, segundo grupo e tempo

Grupos/variáveis	IDNT (n=19)		IDT (n=29)		Aumento da força máxima (grupo IDT) %
	Pré	Pós	Pré	Pós	
Membros inferiores (kg)					
Extensão de joelhos (sentado)	8,05±2,04	8,85±2,58	11,65±2,69	16,68±3,04* (p<0,001)	43,2
Flexão de joelhos (deitado)	7,55±0,51	7,55±1,57	8,19±1,76	13,52±2,25* (p<0,001)	65,1
Membros superiores (kg)					
Supino sentado máquina	20,85±3,60	21,35±3,72	18,58±2,62	23,74±2,82* (p<0,001)	27,8
Remada sentado	22,10±4,27	22,75±3,58	19,19±3,89	25,13±4,43* (p<0,001)	31
Tríceps *pulley*	21,00±4,97	21,95±5,04	20,16±2,19	24,42±2,68* (p<0,001)	21,1
Bíceps *pulley*	6,55±1,76	7,05±1,61	5,45±0,96	7,84±1,32* (p<0,001)	43,9

*Diferença entre condição pré e pós-treinamento (p<0,001). Dados expressos como média ± desvio padrão. IDNT: idosos diabéticos não treinados; IDT: idosos diabéticos treinados.

## DISCUSSÃO

Como tentativa de intervenção e melhora da saúde, especialmente do aumento de força máxima, podendo refletir em mudanças positivas ao organismo, como aumento da autonomia e independência, e mudanças metabólicas,^(18)^ utilizou-se, no presente estudo um protocolo de TRO, com mudança de intensidade e volume semanal. Em se tratando de idosos diabéticos, os aumentos médios de força para cada grupo muscular avaliado no estudo foram bastante significativos.

O maior aumento médio de força foi observado para o movimento de flexão dos joelhos (65,1%), seguido pelos movimentos de extensão dos joelhos e bíceps *pulley* (43,2% e 43,9%), respectivamente. Os movimentos de extensão e flexão de joelho são fundamentais nas atividades da vida diária, para agachar, levantar e em deslocamentos, sendo de grande importância para essa população. O aumento de força nos músculos envolvidos no movimento de flexão do cotovelo (bíceps *pulley*) de 43,9% em relação à condição pré-treinamento pôde auxiliar os indivíduos na realização de tarefas cotidianas e de trabalho que exijam o uso dos membros superiores como, por exemplo, transporte de uma sacola de compras no supermercado, estender e tirar roupas do varal, carregar objetos etc. Parece que esse aumento da força dos músculos flexores do cotovelo está relacionado ao fato de que os músculos bíceps braquial, braquial e braquiorradial participam do movimento tanto no exercício de bíceps *pulley* quanto de remada sentado, o que pode justificar ao menos em parte esse maior ganho médio de força.

Os efeitos do processo de envelhecimento, associados à inatividade física, atuam de forma diferente nos membros superiores e inferiores. O declínio de força parece ser mais acentuado nos membros inferiores.^(27-30)^ Dessa forma, espera-se que, para indivíduos sedentários, a resposta frente a um treinamento físico de força seja mais evidente nos membros inferiores, uma vez que estes estão menos treinados. Como participaram do presente estudo idosos não fisicamente ativos, essa resposta do treinamento foi observada, revelando um aumento de força maior para os membros inferiores.

Nos demais exercícios realizados, também foram verificados aumentos médios de força para o movimento de supino reto (27,8%) e tríceps *pulley* (21,1%). Tal fato demonstra que o treinamento físico desenvolvido foi eficiente em aumentar a força dos participantes. No entanto, esperava-se um aumento até mais acentuado dessa variável para o movimento de extensão de antebraço, uma vez que, no exercício de supino reto e de desenvolvimento com halteres, o músculo tríceps também participa da realização do esforço, havendo, então, uma somatória de recrutamento dessa musculatura no movimento.

A particularidade deste estudo esteve na proposta de um protocolo de TRO, na frequência de três vezes por semana, com intensidade e volume diferentes em cada semana, realizadas de forma periódica (aumento de carga). Esse tipo de treinamento com objetivo de aumento de força máxima em idosos diabéticos tem sido pouco explorado na literatura. Quando comparado aos diversos protocolos de treino físico de força utilizados em indivíduos diabéticos do tipo 2, o treinamento físico aqui proposto se mostrou bastante eficiente na promoção de aumento de força.^(8-12)^ Estudos da literatura, realizados com outras populações (crianças e adultos jovens), também têm observado respostas mais significativas na força máxima e parâmetros metabólicos (por exemplo: sensibilidade à insulina) com o TRO em relação ao treinamento resistido linear.^(17,21)^

Em nosso estudo, o aumento médio de força dos membros inferiores foi de 54,15% e de membros superiores 30,95% em 16 semanas de treinamento físico, sendo esses resultados similares ou superiores aos encontrados na literatura.^(8-12,23)^ No entanto, é preciso mencionar que tal fato pode estar relacionado à característica da amostra, que foi constituída de idosos diabéticos, não fisicamente ativos e não familiarizados com o exercício físico resistido. De acordo com os princípios do treinamento, os indivíduos fisicamente treinados (ou seja, altamente condicionados) apresentam menores respostas adaptativas a um programa de exercício físico.^(31)^

Dunstan et al.,^(32)^ a partir de um treinamento físico resistido de cargas progressivas, iniciando com 50% e terminando o período de treinamento com cargas atingindo 80% de 1RM, com duração do programa de exercício físico por 6 meses, obtiveram resultados semelhantes aos encontrados neste estudo, quanto ao ganho de força em idosos diabéticos. Entretanto, diferente do presente estudo, os ganhos maiores de força foram obtidos nos membros superiores dos idosos diabéticos treinados.^(32)^ Por sua vez, Cauza et al*.*,^(14)^ ao empregarem um programa de treinamento físico resistido com aumento de intensidade e volume de modo progressivo das cargas de trabalho com duração de 16 semanas, em uma população de adultos com DM2, também observaram boas respostas do treinamento de força nessa população específica. Similarmente aos resultados do presente estudo, Cauza et al. também observaram maiores aumentos de força nos membros inferiores, quando comparados aos superiores.^(14)^

Em outro estudo, também com uma população de idosas diabéticas, com idade média de 68 anos, Guido et al. obtiveram resultados semelhantes aos do presente estudo, observando que o treinamento resistido de 24 semanas, com intensidades progressivas a cada 4 semanas, iniciando em 60% até atingir 80% de 1RM, foi capaz de aumentar a força de membros inferiores, principalmente dos músculos extensores do joelho.^(33)^ Embora os resultados obtidos na literatura apontem aumentos de força com o treinamento linear, um dos aspectos positivos observados no TRO é que a execução (rotina) dos exercícios se torna menos monótona e, portanto, há maior assiduidade do participante ao programa.^(16)^

O aumento na força obtida com o treinamento físico resistido no presente estudo pode repercutir em melhor qualidade de vida e em autonomia dos participantes. A força é uma capacidade física muito importante, que, quando aumentada, proporciona também melhora de outras capacidades, como a agilidade e equilíbrio, o que é extremamente importante para evitar acidentes domésticos em indivíduos da terceira idade.^(34)^ Ferreira e Gobbi^(35)^ verificaram que mulheres idosas ativas, que consequentemente possuem melhores níveis de força, apresentaram melhores níveis de agilidade de membros inferiores, quando comparadas às mulheres idosas sedentárias.

Levando em consideração que com o envelhecimento ocorre do fenômeno chamado de “sarcopenia”, que é a diminuição da massa muscular, e que a capacidade do músculo em gerar força em humanos declina especialmente após os 60 anos,^(36)^ pode-se dizer que o treinamento resistido realizado pelos participantes contribuiu para retardar esse processo, proporcionando ganhos adicionais de força ao longo de 16 semanas. Contudo, são necessários novos estudos com pessoas diabéticas ativas e por um período maior de tempo, para que seja possível avaliar os resultados do programa aqui proposto.

## CONCLUSÃO

De acordo com a amostra estudada e levando em consideração as limitações do presente estudo (menor número de indivíduos do sexo feminino no grupo de Idosos Diabéticos Não Treinados em relação ao grupo de Idosos Diabéticos Treinados, além do período curto de intervenção – 16 semanas), pôde-se concluir que o protocolo de treinamento físico resistido ondulatório utilizado mostrou-se eficiente em proporcionar significativos aumentos de força máxima, tanto de membros inferiores, quanto de membros superiores em indivíduos diabéticos do tipo 2 não fisicamente ativos. Sugere-se que o programa aqui proposto possa ser utilizado como alternativa diferenciada no treinamento de força para população idosa diabética do tipo 2 e, sobretudo, no que diz respeito aos profissionais da área da saúde que atendem a essa população específica.
